# The Role of One’s Motive in Meditation Practices and Prosociality

**DOI:** 10.3389/fnhum.2019.00048

**Published:** 2019-02-13

**Authors:** J. Shashi Kiran Reddy, Sisir Roy

**Affiliations:** ^1^Research Scholar, Bangalore, India; ^2^National Institute of Advanced Studies, Indian Institute of Science (IISc) Campus, Bangalore, India

**Keywords:** meditation, prosocial, motive, compassion, intent, mindfulness, imagination

## Abstract

No individual exists without exhibiting prosociality in one or another situation during their lifetime. The argument however, is to what extent? Does it arise spontaneously, out of true empathy and compassion for others, or it is goal-oriented with some hidden motive? Here, our primary intention is to convey that, though various meditation-based interventions can be utilized for different purposes like cultivating prosocial behaviors such as compassion, empathy etc., one’s underlying motive and intent seems to play a crucial role in an individual’s development. Most of the studies exploring prosociality, in the context of meditation, usually do not consider the role of hidden or underlying motivation in one’s prosocial expression. By considering an example of how mindfulness may sometimes lead to the wrong consequences, we try to analyze why it is important to include the aspect of inner motivation in future studies exploring the effects of meditation on prosociality. We also propose that while practicing meditation one may need traditional assistance and ethical/moral teachings in addition to those merely isolated techniques.

## Introduction

It is natural that we question the nature of the world we perceive, but we also question the very nature and purpose of life. Thus, many ancient cultures and civilizations across the globe have already put forward and proposed that the social wellbeing of all and of the self is the goal of an individual life (Neusner and Chilton, 2005). In addition, these cultures even developed various techniques and practices of meditation, capable of cultivating prosociality (Monteiro et al., 2015; FitzPatrick, 2017; Kirby and Gilbert, 2017; Luberto et al., 2018). The set of behaviors that involves the concern and helpful feelings for others, are commonly defined as prosocial (San Martín et al., 2016; FitzPatrick, 2017; Goetz and Simon-Thomas, 2017). At times, these behaviors are expressed even at the cost of self-suffering or personal benefit. In general, apart from the internal motivation caused by the feelings of empathy, it also essentially involves transcending one’s selfishness (Saslow et al., 2013). Therefore, prosociality is usually associated with social behaviors such as compassion, kindness and altruism. The extent and type of prosocial expression of an individual, may also vary as per the situation and the nature of the interaction.

In the present context, it is meaningful to ask- is prosociality an inherent nature of one’s social expression? Or does it have to be cultivated only through external means? The question is if it is innate, why should one practice it by other means? If so, what does it take for us to realize that prosocial expression is a natural quality? A recent study also supports the idea that we are all altruistic (selfless) at heart and that it takes some effort to be selfish; which means that altruism is our default social expression (Christov-Moore et al., 2017). Currently, we only have preliminary evidence supporting such claims and we need more studies to explore the evolutionary and functional basis of prosociality. Although prosocial behaviors are mostly studied in the context of social psychology and evolutionary psychology, recently some studies have attempted to explore these behaviors in relation to meditation.

In recent times, because of the popularity of different practices of meditation; mainly mindfulness and mindfulness-based interventions (MBIs), there is growing interest in studying not only the effects of meditation on an individual (focused on isolated physical and psychological effects) but also its social influence (mainly interpersonal and collective effects). In order to explore the ability of meditation for prosociality, we now have numerous studies quoting the benefits of practicing several techniques like mindfulness, compassion and loving-kindness meditation (LKM) and other meditation-based interventions (Hofmann et al., 2011; Gilbert and Choden, 2013; Gilbert, 2014; Kirby et al., 2017; Matos et al., 2017; Luberto et al., 2018). But, as these studies indicate, though one may develop prosocial behaviors like compassion and altruism by means of certain meditation practices, conventionally cultivating prosociality alone does not seem to be the essential goal of meditation. Accordingly, all these claimed aspects are epiphenomenal, resulting from practicing different meditation techniques (Gilbert and Choden, 2013).

Nowadays, we reduce meditation as just a tool or technique for cognitive enhancement and wellbeing. Subsequently, different practices of meditation are treated as a set of relaxation techniques and stress relieving methods. Some even propose meditation as a practice for enhancing various cognitive functions based on the evidence that it helps to focus ones attention, concentration, and heightens cognitive inhibition (Ospina et al., 2007; Rao, 2011; Schmidt and Walach, 2014; Tang et al., 2015; Coronado-Montoya et al., 2016; Luberto et al., 2018). There are also others who use various meditation techniques as alternate methods of healing, to replace conventional practices, or at times in association with other treatment methods.

Although some acknowledge the benefits of meditation practices, there are others who argue that the contemporary view of meditation deviates from the traditional understanding (Rukmani, 2001; Rao, 2011; Awasthi, 2013; Nash and Newberg, 2013; Monteiro et al., 2015; Reddy and Roy, 2018). They indicate that different practices of meditation, though they may be beneficial, have an essentially different purpose. They propose that to study meditation in its extent and entirety, one would also need to be aware of its traditional significance and the teachings that accompany these practices. Here, one must note that meditation has a higher purpose, and different techniques have been devised for the purpose of self-realization and enlightenment, and for understanding the nature of one’s own mind (Rukmani, 2001; Rao, 2011; Schmidt and Walach, 2014; Reddy and Roy, 2018).

Even when considering meditation as just a tool, without any traditional aspects, we already have numerous studies investigating the role of meditation for different purposes. Thus, it is a reasonable attempt to develop a consistent overview of various claims put forth by different scientific studies on meditation. The major focus of these studies, apart from exploring meditation as a tool for wellbeing and alternative clinical intervention, is to study its potential to cultivate prosociality. Many studies have focused on meditations ability to develop prosocial behaviors like compassion, kindness etc. Since some studies claim to show evidence that certain meditation-based interventions can be used to cultivate prosociality, there is a need to review and analyze these studies to see if such claims are true. Thus, the focus in Kreplin et al. (2018) was to systematically review and meta-analyze the effects of these practices on prosociality in randomized controlled trials (RCTs) of healthy adult subjects. Although the authors agreed with meditation’s potential to influence prosociality to some extent, they were skeptical of various overstated claims and popularity. They noted that most of the concerned studies presented a very tenuous and unclear justification as to why a meditation intervention ought to improve prosocial outcomes.

Kreplin et al. (2018) authors even indicated that meditation seems to only have a limited influence on prosocial behaviors; mainly towards the feelings of compassion and empathy. Their findings also suggest that these prosocial effects, at least in part, may be a result of methodological frailties involved in the concerned studies, for instance, biases introduced by the meditation instructor, the type of control group selected and the beliefs and expectations of participants about the power of meditation. Therefore, they offer potential ways to overcome these issues to some extent in future studies.

In addition, while studying social behavior, another significant aspect which needs critical attention is the underlying motive and intent (Hirschberger and Lifshin, 2013). The role of underlying motivation in one’s prosocial expression has received very little attention in studies concerned with the effects of meditation on prosociality. If we analyze carefully, an individual’s hidden motivation and intention can reduce a social interaction to a goal-oriented task which also applies to prosocial behaviors like compassion and empathy. Considering the aspect of inner motivation as a defining factor, here, we propose that some practices of meditation, without a rightful intent and motive, may be incomplete and when addressing these aspects, traditional knowledge and insight might be helpful (Ricard, 2009; Rao, 2011; Monteiro et al., 2015; Reddy and Roy, 2018). This means that neither practicing different meditation techniques by themselves nor their potential to cultivate prosocial effects make them wholesome and complete in the absence of moral teachings. Although some scientific studies on meditation seem to lack traditional understanding, it is important to note that meditation as a practice (without being associated with any tradition) shows many benefits for various purposes. Thus, currently, it is being explored for different clinical and psychological purposes.

## The Role of Right Motive and Intent in the Practice of Meditation

Nowadays, meditation is promoted mainly as the practice of wellbeing. Since numerous studies attribute various benefits to the practice of meditation, many individuals are inclined to learn and practice meditation techniques (Kreplin et al., 2018). It therefore creates high expectations among participants, which creates the need for more meditation courses and retreats. While it is a good sign that many people want to practice and benefit from these ancient techniques, an individual can truly merit only when he/she is well aware of the traditional understanding of these practices and their consequences (Monteiro et al., 2015). This is because most of the devised techniques are not isolated practices (not complete practices by themselves), and should be combined and supplemented with a specific lifestyle and moral teachings (Ricard, 2009; Monteiro et al., 2015). Here, the ethical component comes into the picture. For a detailed argument, let us consider “mindfulness practices.” These practices usually involve some form of meditation, with the goal to create and maintain a non-reactive and non-judgmental state of focused awareness. These techniques can still be exercised independently of spiritual motive. Thus, most researchers and traditional practitioners believe that mindfulness in the absence of traditional knowledge and ethical teachings may sometimes lead to the wrong consequences (Ricard, 2009; Monteiro et al., 2015; Kreplin et al., 2018).

For instance, a sniper whose intention is to kill another human being would be as mindful as a meditator of his body, thoughts, and feelings before pulling the trigger (Ricard, 2009; Kreplin et al., 2018). While he/she acts mindfully in this situation, it is the intention and inner motivation which matters. It shows that mindfulness by itself, as a focused state of attention, is neither good nor bad. As specified by a well-known Buddhist monk Matthieu Ricard (2009)

“*bare attention, as consummate as it might be, is no more than a tool that can certainly be used to achieve enlightenment and is needed for this purpose, but which can also be used to cause immense suffering. Obviously, what is entirely missing is the ethical dimension of a mindfulness that deserves the qualification of ‘wholesome’ and can lead to enlightenment*”

He also stated that genuine mindfulness teachings should not only include a means to direct and maintain one’s attention on the chosen object, but also the understanding of the nature of the mind and one’s own mental state. In addition, these teachings should also embed an ethical element which enables one to clearly distinguish if it is helpful to maintain the present state of mind and behavior. Thus, it appears that mindfulness should be practiced with the aspiration to achieve enlightenment for the welfare of oneself and for all beings (Ricard, 2009; Monteiro et al., 2015).

In addressing such claims, some researchers propose that one should consider meditation only for its evidence-based benefits, with ethical and moral aspects being independent attributions of different cultural biases and ethical dogmas (Coronado-Montoya et al., 2016; Klein and Epley, 2016; Kabat-Zinn, 2017; Mattes, 2018). Here, they consider moral behaviors to be independent of these practices. They are acquired not through practice alone but by means of the induced cultural dogmas. Accordingly, the sniper’s analogy (in connection to the moral behavior and mindfulness) cannot simply be used without the context, as we have various instances in the same culture where highly revered spiritual masters have killed their enemies to maintain dharma (or righteousness) in the world. Addressing the context, in Mattes (2018), the author states that—“*More mindfulness of our human cognitive limitations should lead to less dogmatism in general, and in ethical matters in particular.”* In the current situation, and simply based on the preliminary evidence, it is tough to say if prosocial abilities attributed to meditation is because of the practices alone or because of the supplemented traditional teachings. Such a connection has not been explored in detail and thus we propose that more studies on meditation and its potential to develop prosocial behaviors in the context of moral and ethical dogmatism, in a particular culture, are needed. The role of traditional teachings in cultivating prosocial behaviors should therefore also be studied in depth.

## Underlying Motive and Its Role in Prosocial Behavior

In the modern world, we have reached a state where we make ourselves and our behaviors so encrusted and complex that it becomes tough to comprehend. In some instances, it is hard to know why people behave the way they do because their true motives remain hidden (Hirschberger and Lifshin, 2013). The conditions where motives drive a specific behavioral choice are quite possible. For example, there are many instances in life where we should take one or the other decision. Although every decision we make is intended to benefit us, sometimes it is true that we make decisions for others in order to help them. Here, our prosocial nature enters the picture. It is more important when we are dealing with matters where others are involved. The extent and type of prosocial expression may vary according to the situation. In some cases, while few individuals may express such behaviors only during conditions where others are in trouble or pain, others try to cultivate a behavior which is spontaneously selfless in all conditions.

Just like the possibility of cultivating mindfulness for the wrong purposes, one may also develop wrongful prosocial behaviors; acting prosocial with the wrong motives and intent. On the fringe, one may act compassionate and look altruistic, but how reliable is it? We need a way to identify if altruistic behavior of an individual is prompted by one’s selfish motives and goal-oriented thoughts or by true empathy and compassion. In the present context, it is central to study and understand not only the behavior of an individual, but also the motive and intent behind any such conduct (Hirschberger and Lifshin, 2013). It is surprising to see that none of the previous studies that explored the effects of meditation on prosociality take note of inner motivation, which is crucial when it comes to the application of practices or teachings that one learns. Therefore, different practices of meditation and various other interventions, which are known to assist in cultivating prosocial behaviors, should consider the hidden or underlying motivation.

In addition, as there are various meditation practices across different traditions, it is not true that every meditation technique will cause the same influence on prosociality. Leaving prosociality, the regions of the brain affected by different meditation techniques are also dissimilar. Studies show that each meditation technique will have a unique neural impact associated with a particular practice. In a recent meta-review study (Fox et al., 2014), 78 functional neuroimaging studies on meditation practices were examined. The findings suggest a clear neurophysiological dissociability among different meditation practices, in addition to some methodological issues and concerns. They also establish that although several brain regions may be equally affected in various meditative practices, differences greatly outnumber the existing similarities. So, they state that “commonality across meditation categories is the exception rather than the rule.” In another meta-review, Lee et al. (2018) examined the neural oscillations that underlie meditation. They also noticed some significant changes in the neural oscillatory activity among different meditation practices, in agreement with Fox et al. (2014). They also indicate that more rigorous studies are needed to elucidate the nuanced imaging and physiological changes that occur with each type of meditation. In addition to the distinct physiological and psychological changes associated with different meditative practices, another important aspect to study and identify is inner motive and intent. This aspect plays an important role in prosocial behavioral studies. We therefore need to study and understand the neural basis in order to distinguish between our inner motivations and intentions.

Considering the functional complexity of the brain, it is truly tough to indicate a specific region in the brain connected with one’s inner motive and intent. Nevertheless, it is interesting to see that some recent studies have attempted to delineate various neural patterns connected to these aspects (Hein et al., 2016; Cutler and Campbell-Meiklejohn, 2019). A recent study showed that, if some active brain regions can give information regarding one’s behavior, then studying how the respective brain areas interact helps in understanding the motive behind such behavior (Hein et al., 2016). Which implies that one’s motives can be distinguished from each other, as they are characterized by a specific interplay between different brain regions. Another study reported, for the first time, the way in which to differentiate between the motives of individual subjects (Cutler and Campbell-Meiklejohn, 2019). They showed how to split the mechanisms between what happens in the brain when we act out of genuine altruism—where there is no personal benefit, and when we express a strategic kindness—with the intention to personally gain or benefit as a consequence.

Such findings can contribute effectively to knowing the extent and role of one’s hidden motive in future studies on compassion and altruism. It also provides a reliable foundation/base for studying the efficacy of various practices of meditation. Even though research into finding the underlying neural mechanisms and correlates are in preliminary stages, such studies would help us better understand the areas associated with our inner motivations and intentions. We therefore appreciate these studies and call for more research on connecting these aspects. These studies will be helpful not only in the context of meditation research, but with prosociality in general.

## The Potential Influence of Motive on Mental Faculty in Cultivating Prosociality

If we review different meditation practices and other interventions used to cultivate prosocial behaviors like compassion and empathy, we find that most make use of one’s capacity to imagine. This is because, a person’s capability to imagine them self in another person’s situation or condition forms the very basis of empathy and compassion (Gilbert and Choden, 2013; Matos et al., 2017). On the other hand, if imagination can assist in cultivating prosocial behaviors, there is a possibility that the same can easily be applied to develop antisocial behaviors. That means the same technique can also be used to develop a negative attitude towards others. So, what is significant is the ethical sense and traditional knowledge, rather than the simple techniques which employ the use of cognitive faculties such as imagination and visualization etc. As specified in Kreplin et al. (2018), sometimes, one may cultivate prosociality not truly because of practicing these techniques, but through the expectation factor which motivates one to learn. So, meditation in no way looks different from other techniques that simply help in developing focused cognitive enhancement. Even in the above context, it is very important to see and study what really matters, whether the practice itself can cultivate a prosocial behavior or if it is a result of the supplemented ethical teachings.

## Future Directions for Studies on Meditation and Prosociality

In addition, like in the Buddhist tradition, philosophical schools of Hinduism also emphasized prosocial aspects such as compassion and altruism. Although we have some preliminary evidence from recent studies that meditation affects prosocial behaviors, the main focus of the majority of these studies were on Buddhist contemplative practices (Hofmann et al., 2011; Gilbert and Choden, 2013; Gilbert, 2014; Kirby et al., 2017; Matos et al., 2017; Luberto et al., 2018). Since exhibiting these prosocial aspects is the fundamental and essential teaching in different schools of Hinduism, we need scientific studies that investigate the role of these practices in cultivating empathy, compassion and altruism etc. Along these lines, one can also have a comparative study on the potential influence of different practices that emanates from different spiritual traditions.

As discussed earlier, we have two groups of researchers, one group argues that meditation should be considered solely for its evidence-based benefits and the other group claims that the contemporary view of meditation is different from the traditional view. Thus, the latter group argues that the role of traditional aspects has to be considered in meditation research. Findings of Kreplin et al. (2018) show that what we consider evidence—meditation practices being helpful in developing prosociality—suffers from various biases and issues, and thus cannot be regarded as true evidence. We need more scientific studies with a better methodological design and conceptual framework to avoid such biases. In light of various such perspectives, future studies on meditation and prosociality should address the following possibilities: (i) different biases and issues associated with the studies concerned with meditation’s influence on prosocial behaviors; (ii) the role of ethical and moral teachings and other traditional aspects in cultivating prosocial behaviors in the context of meditation; and (iii) the influence of one’s inner motives and intent on prosocial behaviors.

## Discussion

“*It is quite true that a meditator resting in pure awareness and perfect understanding of the fundamental nature of mind, unaltered by mental constructions, will be unable to pull the trigger and kill someone. This kind of luminous awareness is a state of wisdom and is the natural state of a mind that is entirely free from ignorance and mental toxins and spontaneously imbued with unconditional altruism and compassion. Such a state is the result of having achieved inner freedom and should not be confused with mere mindfulness and bare attention”* (Ricard, 2009).

The above quote describes the mental state of a meditator who has achieved the goal of meditation as indicated by the cultural tradition. Considering the sniper example, it states that a meditator with genuine kindness and who is well aware of the nature of the mind, will not be able to pull the trigger to kill someone. It is because he/she is not only resting in a state of perfect mindfulness but is also aware of the perturbations of the mind that may result in immoral acts. Here, we are not sure if such an understanding results from meditation practices or from supplemented traditional teachings. Thus, it is always important to study the role of traditional teachings and cultural claims in the context of meditation and prosociality.

## Concluding Remarks

In the present scenario, where there is a sharp decline of moral and ethical values across the globe, causing individual and social degradation in all possible forms, it is essential to investigate different possible ways to cultivate prosocial behaviors. In light of this, it is important to examine the potential of different contemplative practices, like meditation, to cultivate prosocial behaviors. Since some studies suggest that meditation can bring about long- and short-term functional, physiological and psychological changes, it is essential to explore meditation as a means to develop prosociality.

Exhibiting different prosocial emotions and behaviors is important for both individual and societal well-being. This is one reason why practicing and exhibiting prosocial behaviors such as empathy, kindness and compassion has been emphasized across diverse social institutions and major religions of the world. The outcome of prosociality has a positive impact on public health because it not only benefits the individual receiving help, but also the individual offering the help. This aspect of prosociality has been emphasized in Hindu and Buddhist philosophical systems as well—where they state that, by helping others, an individual is actually helping them self. Indeed, this traditional understanding is now supported by some research studies that demonstrate that engaging oneself in prosocial behavior is not only associated with greater happiness and psychological well-being but also has other indices of physiological health.

Studies exploring the role of meditation in cultivating different prosocial behaviors like empathy, kindness, compassion and altruism have received much attention lately. Although, meditation includes a diverse set of practices across different traditions, these studies mainly focus on Buddhist contemplative practices such as mindfulness, vipassana, LKM etc. On one side, a large body of studies suggest that meditation practices influence prosociality to a greater degree, whereas on the other side, some researchers indicate that the majority of the claims regarding the prosocial effects of meditation are overstated and could be a result of various issues associated with the methodology and design of the respective study.

Therefore, in this article, we examined some issues related to the studies associated with meditation and prosociality. We emphasize one’s inner motive and intent as a crucial aspect to take into consideration in prosocial studies. In addition, our primary purpose here is to highlight the role of traditional knowledge and the ethical teachings associated with meditation practices; mainly mindfulness and its based interventions, in cultivating prosociality. We also indicate that, in order to study and examine the ability of meditation practices to develop prosocial behaviors in an individual, we need more studies with a clear methodology and research design as well as studies across different spiritual traditions. We also offer some direction for future studies on meditation and prosociality.

## Author Contributions

JR came up with an idea to write the manuscript. Both JR and SR analyzed and wrote the article.

## Conflict of Interest Statement

The authors declare that the research was conducted in the absence of any commercial or financial relationships that could be construed as a potential conflict of interest.
